# Burkitt lymphoma: The effect of age, sex and delay to diagnosis on treatment completion and outcome of treatment in 934 Patients in Cameroon

**DOI:** 10.1371/journal.pone.0299777

**Published:** 2024-03-11

**Authors:** Peter Bernard Hesseling, Glenn Mbah Afungchwi, Bernard Wirndzem Njodzeka, Paul Wharin, Francine Nicole Kouya, Mariana Kruger

**Affiliations:** 1 Department of Paediatrics and Child Health, Stellenbosch University, Cape Town, South Africa; 2 Department of Nursing and Midwifery, The University of Bamenda, Bamenda, Cameroon; 3 Cameroon Baptist Convention Health Services, Bamenda, Cameroon; 4 Beryl Thyer Memorial Africa Trust, Burton Latimer, United Kingdom; University of Ottawa, CANADA

## Abstract

**Introduction:**

The role of age and sex in the presentation and outcome of endemic Burkitt lymphoma (BL) has not been studied recently. This study analysed these factors in 934 patients with BL who had received cyclophosphamide and intrathecal methotrexate as treatment.

**Methods:**

Records of 934 children diagnosed with BL from 2004 to 2015 were obtained from our Paediatric Oncology Networked Database (POND) cancer registry. Age at diagnosis, sex, disease stage, time to diagnosis, delay in diagnosis, completion of treatment, rate of abandonment, and one-year survival rates were recorded and statistically analysed.

**Results:**

The male to female ratio of 1.41 for the study population of 934. The median delay from onset of symptoms to diagnosis was 31 days. The St Jude stage distribution was I = 6.4%, II = 5.9%, III = 71.5% and IV = 16.2%. Significantly more patients presented with stage III disease in age groups 5–9 and 10–14 years than 0–4 years. The overall 1-year survival rate was 53.45%, respectively 77.1% for stage I, 67.9% for stage II, 55.1% for stage III and 32.4% for stage IV disease (p<0.001). There was no significant difference in survival by sex and age group.

**Conclusion:**

Patients aged under 5 years presented with less-advanced disease, but survival was not affected by age. Sex did not influence delay to diagnosis and overall survival. The long delay between the onset of symptoms and diagnosis emphasises the need for interventions to achieve an earlier diagnosis and a better survival rate.

## Introduction

Burkitt lymphoma (BL) is a significant problem for childhood cancer in many countries in Africa, with an estimated number of 3900 new cases in 2018, with 81% occurring in the age group 0–15 years [1]. The highest incidence rate is found in Sub—Saharan countries with and average incidence rate per 100,000 children aged 0–14 years of 0.86 in males and 0.42 in female, and an age standardized incidence rate (ASR) of 0.35 in males and 0.20 in females [1–4]. An ASR of 4.6 and 3.07 was recorded in the capital Yaounde and in the Northwest region of Cameroon respectively [5,6]. The male: female ratio of 1.5 and median age at diagnosis of 7.9 years in BL patients in Northwest and Southwest Cameroon is similar to that in other African countries [4]. A numerical predominance in incidence has been reported for males over females, as well as the highest incidence in the age group 5–9 years in Cameroon and elsewhere [3,5,6].

BL is one of the six common and curable cancers identified for increased focus by the World Health Organization global initiative for childhood cancer, an initiative launched in 2018 that aims to increase the global survival rates of childhood cancer to 60% globally by 2030 [7]. Cure rates of over 80% are the norm in high-income countries [8]. In low-income sub-Saharan African countries with limited resources, the recorded cure rate is much lower, but a cure rate of 50–60% is possible with low-cost, risk-adapted protocols that include free treatment and parental support [9–11]. In Northwest Cameroon, parents of patients diagnosed with BL with a low socio-economic score however achieved the same cure rate as patients with an improved socio-economic status when free treatment, nutritional support and parental support were provided to all, except in patients of single mothers who had poor access to family and community support [12].

The role of age and sex on the presentation and outcome of treatment was studied in 387 BL patients in Ghana for the period 1966–1978, and showed no influence of age and sex on survival [7]. Staging was however different to the St Jude staging in current use, and although all patients received cyclophosphamide in different dose schedules, the same treatment protocol was not used in all patients. In a cohort of 2084 children and adolescents with various forms of non-Hodgkin lymphoma in Germany, survival was better in males with T–lymphoblastic lymphoma and diffuse large B-cell lymphoma and patient in 0–4 years age group had inferior event-free survival for precursor B-cell lymphoblastic lymphoma and anaplastic large cell lymphoma [13]. Age and sex at diagnosis have not previously been recorded to be a risk factor for survival for Burkitt lymphoma [14]. On the other hand, delays to diagnosis has been reported as an important contributor to poor outcomes of various childhood cancers in low- and middle-income countries [15]. This has been shown to poorly affect outcome for retinoblastoma in Cameroon [16]. The aim of this study was to investigate the effect of age at diagnosis and sex and delays to diagnosis on outcome in a large cohort of patients with BL who received standard cyclophosphamide-based chemotherapy protocols at three treatment centres in rural Cameroon.

## Methods

This was a retrospective descriptive study that included all patients who had been admitted to Banso and Mbingo Baptist Hospitals in the Northwest region of Cameroon and to Baptist Hospital Mutengene in the Southwest region of Cameroon with a diagnosis of BL between 2004 and 2015. Data were retrieved from the Pediatric Oncology Networked Database (POND) electronic registry. Despite limitations of incomplete case ascertainment and missing data, the usefulness of this registry in providing useful data for service evaluation and outcome analysis has been described elsewhere [17,18]. Patient records prior to 2008 were retrospectively entered into POND from paper-based patient records [18]. All patients were treated with standardised cyclophosphamide-based institutional review board-approved protocols [9–11]. The BL treatment programme at these hospitals has an obligatory follow up period of one year. Follow up data beyond one year may be available opportunistically in case of a specific research project or patient visit to the hospital for other reasons. We limited the follow up information for analysis at one year to provide a clear one-year survival graph for all patients. We have added a comment to the text about the follow up period.

All patients admitted with a diagnosis of BL were included in the analyses. Patients were grouped into age categories 0–4 years, 5–9 years and 10–14 years. Data were analysed using IBM SPSS version 25. Frequency distributions were presented for age, sex, basis of diagnosis, stage of disease and status at one-year follow-up. The median delay from onset of symptoms to diagnosis was calculated and disaggregated by sex, age group and disease stage. Approximate date of onset of symptoms was according to parent’s report and this information was routinely collected on first consultation at the treatment centre and recorded in patient case files. Mann-Whitney U test was used to compare differences in median delay to diagnosis by sex while Independent samples Kruskal Wallis test was used to compare differences in mean delay to diagnosis by age group and disease stage. I Survival at 1-year was considered as being alive without disease after one year from the date of diagnosis. Kaplan Meier curves were used to estimate survival up to one year beginning from the date of diagnosis with death, abandonment of treatment, and loss to follow up considered as events. Mantel-cox Log Rank chi-square was calculated and an alpha level of 0.05 was considered for statistical significance. Chi-square test was used to calculate differences in disease stage, treatment completion, death during treatment, and survival at one year between sex. One-way ANOVA test was used to investigate differences in treatment completion, death during treatment, abandonment and survival at one year by age group and disease stage. Spearman correlation test was used to test corelations between disease stage and death during treatment and survival at one year.

This study was a secondary analysis of data previously obtained for other clinical studies that had received ethical approval (IRB2008-4, IRB2011-7, IRB2016-28, Stellenbosch University HREC—S18/08/163, IRB2020-01). Participation in these studies were entirely voluntary and signed informed consent and assent were obtained from patients and parents. No information was collected that could identify the participants during or after the study. A waiver of ethical consent for this study was obtained from the Cameroon Baptist Convention Health Board Institutional Review Board and the Health Research Ethics Committee of Stellenbosch University.

## Results

A total of 934 patients with BL were recorded in the POND registry in the period 2004–2015 at Banso Baptist Hospital (449, 48.1%), Mbingo Baptist Hospital (316, 33.8%) and Baptist Hospital Mutengene (169, 18.1%). The male (546, 58.5%) to female (388, 41.5%) ratio was 1.4:1. The median age at diagnosis was 8 years (IQR 6–10). The median age at diagnosis was 7.0 (IQR 6–10) for males and 8.0 (IQR 6–10) for females (p = 0.408). The age group 5–9 years (566, 61.0%) had the highest incidence followed by the age groups 10–14 years (263, 28.3%) and 0–4 years (99, 10.7%).

The median delay from onset of symptoms to diagnosis was 31 days (IQR: 20–91). The median delay was 33 days (IQR: 21–91.5] for males and 31 days (IQR: 19.75–76.75) for females (p = 0.085). The longest median delay was seen in the age group 10–14 years (34 [IQR: 21–90.5]), followed by 5–9 years (31 [20–91]) and 0–4 years (31 [14–92]) (p = 0.787). There was no significant correlation between delay to diagnosis with age or sex. The method of diagnosis was mainly cytology or histopathology (683, 73.1%) and clinical investigation/imaging (145, 15.5%). The majority of the patients presented with St Jude Stage III disease (641, 71.5%), followed by Stage IV (145, 16.2%), Stage I (57, 6.4%) and Stage II (53, 5.9%). Disease stage was similarly distributed between sex and age groups, except for Stage II disease which was significantly more predominant in males (n = 39, 7.4%) than in females (n = 14, 3.8%) (p = 0.030). There was a weak positive correlation between disease stage and age at diagnosis (Spearman correlation coeff. 0.076, p = 0.023). However, there was no significant correlation between disease stage and delay from onset to diagnosis.

Most of the patients (775, 85.9%) completed treatment, 86 (9.5%) died during treatment and 41 (4.5%) abandoned treatment. Treatment completion was similar between males (n = 461, 87.1%) and females (n = 312, 83.2%), p = 0.208, and also amongst age groups with 77 (84.6%) aged 0–4 years, 478 (86.9%) aged 5–9 years and 218 (84.5%) aged 10–14 years completing treatment (p = 0.606). After a follow up period of 1 year, 485 (53.4%) were alive, 319 (35.1%) had died, 63 (6.9%) were lost to follow up. The majority of children who survived at one year were in the age group 0 to 4 years (52 [55.3%]), followed by 5 to 9 years (298 [54.0%]) and 10 to 14 years (134 [51.7%]) (p = 0.779). The proportion of deaths within one year was similar amongst age groups, with 10–14 years (n = 100, 38.6%), 0–4 years (n = 32, 34.0%), and 5–9 years (n = 186, 33.7%) (p = 0.383) as illustrated in Table 1.

**Table 1:** Stage, treatment status and delay in diagnosis by sex and age groups.

Characteristic	Total N (%)	Sex N (%)			Age Group N (%)			
		Male	Female	Chi-square (P value)	0 to 4 years	5 to 9 years	10 to 14 years	One-way ANOVA F (Pvalue)
**Disease Stage (N = 896)** *								
Stage I	57 (6.4)	38 (7.2)	19 (5.1)	1.548 (0.266)	13 (14.4)	30 (5.5)	13 (5.1)	**5.767 (0.003)**
Stage II	53 (5.9)	39 (7.4)	14 (3.8)	**5.072 (0.030)**	10 (11.1)	28 (5.1)	15 (5.9)	2.493 (0.083)
Stage III	641 (71.5)	366 (69.4)	275 (74.5)	2.747 (0.099)	53 (58.9)	398 (72.8)	188 (73.4)	**3.987 (0.019)**
Stage IV	145 (16.2)	84 (15.9)	61 (16.5)	0.056 (0.854)	14 (15.6)	91 (16.6)	40 (15.6)	0.082 (0.921)
**Treatment Status (N = 897)** *								
Completed treatment	775 (85.9)	461 (87.1)	314 (84.2)	1.588 (0.208)	77 (84.6)	478 (86.9)	218 (84.5)	0.502 (0.606)
Died during treatment	86 (9.5)	47 (8.9)	39 (10.5)	0.626 (0.490)	9 (9.9)	44 (8.0)	32 (12.4)	2.002 (0.136)
Abandoned treatment	41 (4.5)	21 (4.0)	20 (5.4)	0.977 (0.334)	5 (5.5)	28 (5.1)	8 (3.1)	0.899 (0.407)
**Patient Status at 1 year (N = 908)** *								
Alive at one year	485 (53.4)	276 (52.0)	209 (55.4)	1.061 (0.312)	52 (55.3)	298 (54.0)	134 (51.7)	0.250 (0.779)
Died within the first year	319 (35.1)	187 (35.2)	132 (35.0)	0.004 (1.000)	32 (34.0)	186 (33.7)	100 (38.6)	0.960 (0.383)
Lost to follow up	63 (6.9)	47 (8.9)	16 (4.2)	**7.248 (0.007)**	5 (5.3)	40 (7.2)	17 (6.6)	0.256 (0.774)
**Delay in diagnosis in days from onset of symptoms (N = 791)** *	**Total: Median (IQR)**	**Male: Median (IQR)**	**Female: Median (IQR)**	**Mann-Whitney U test P value**	**0 to 4 years Median (IQR)**	**5 to 9 years Median (IQR)**	**10 to 14 years Median (IQR)**	**Kruskal Wallis Test P value**
**Delay in diagnosis**	31.0 (20.0–91.0)	33.0 (21.0–91.5)	31.0 (19.75–76.75)	0.086	31.0 (14.0–92.0)	31.0 (20.0–91.0)	34 (21–90.5)	0.792

*Missing data = 934 –N.

Treatment completion, outcome and abandonment were significantly affected by disease stage. Completion rates were 94% (54/57) for Stage I, 96.2% (51/53) for Stage II, 87.4% (560/641) for Stage III and 73.8% (107/145) for Stage IV (p = 0.000). Abandonment was highest for stage IV (n = 13, 9%), followed by Stage III (n = 25, 3.9%) and Stage II (n = 2, 3.8%), while there was no abandonment in Stage I patients (n = 0, 0%) (p = 0.018).

**Fig 1:**
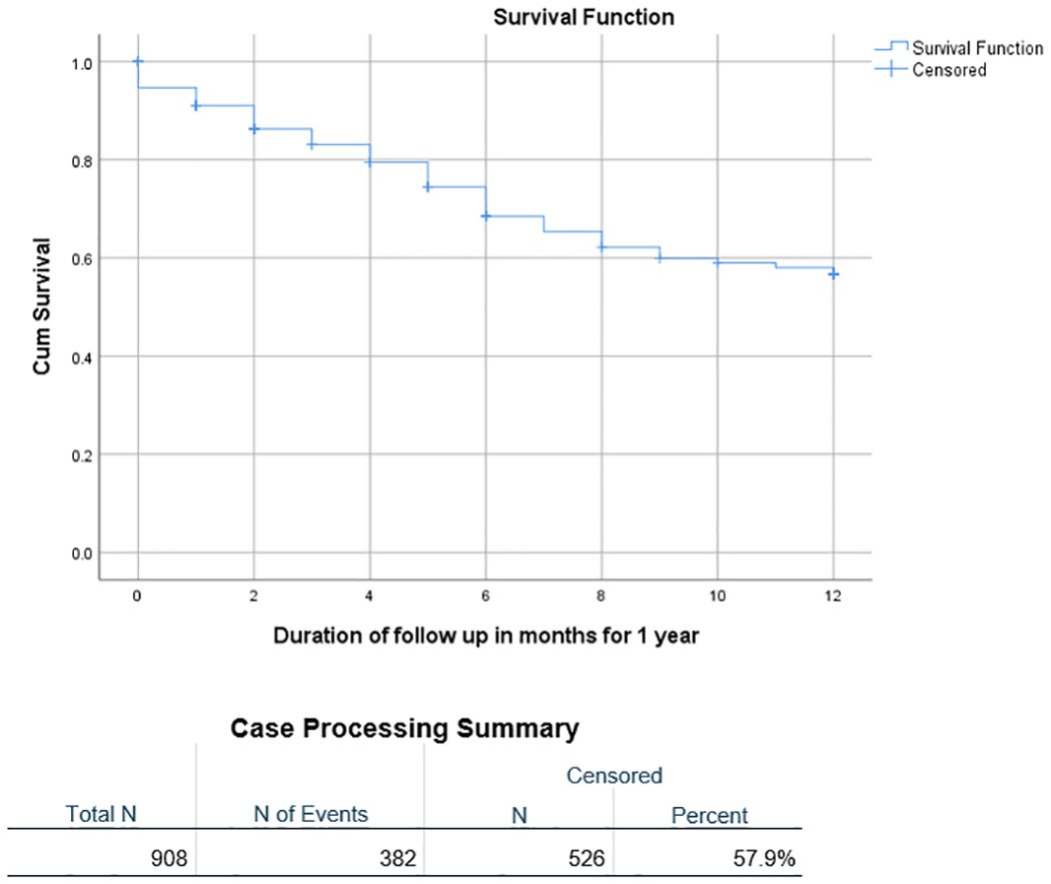
Event-free survival at one year.

**Fig 2:**
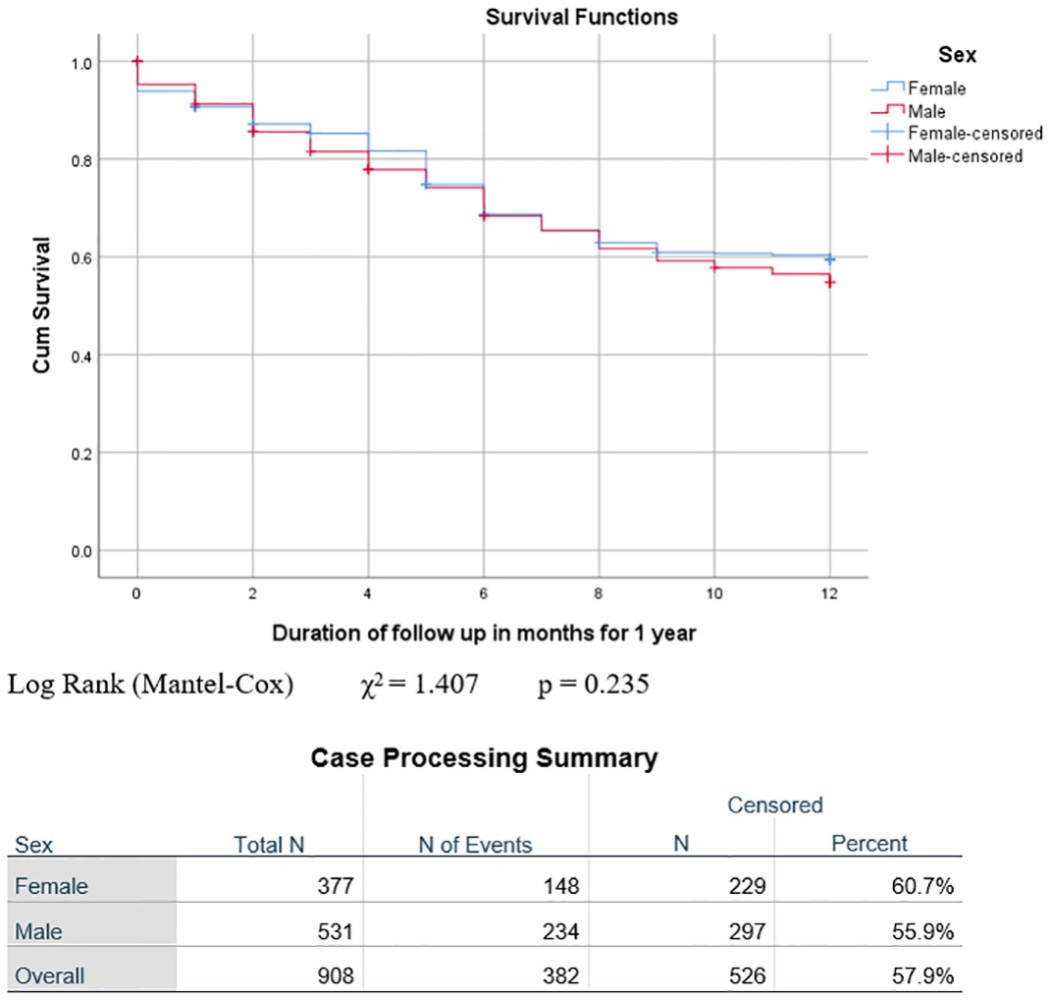
Event-free survival at one year by sex. Log Rank (Mantel-Cox) χ^2^ = 1.407 p = 0.235.

**Fig 3:**
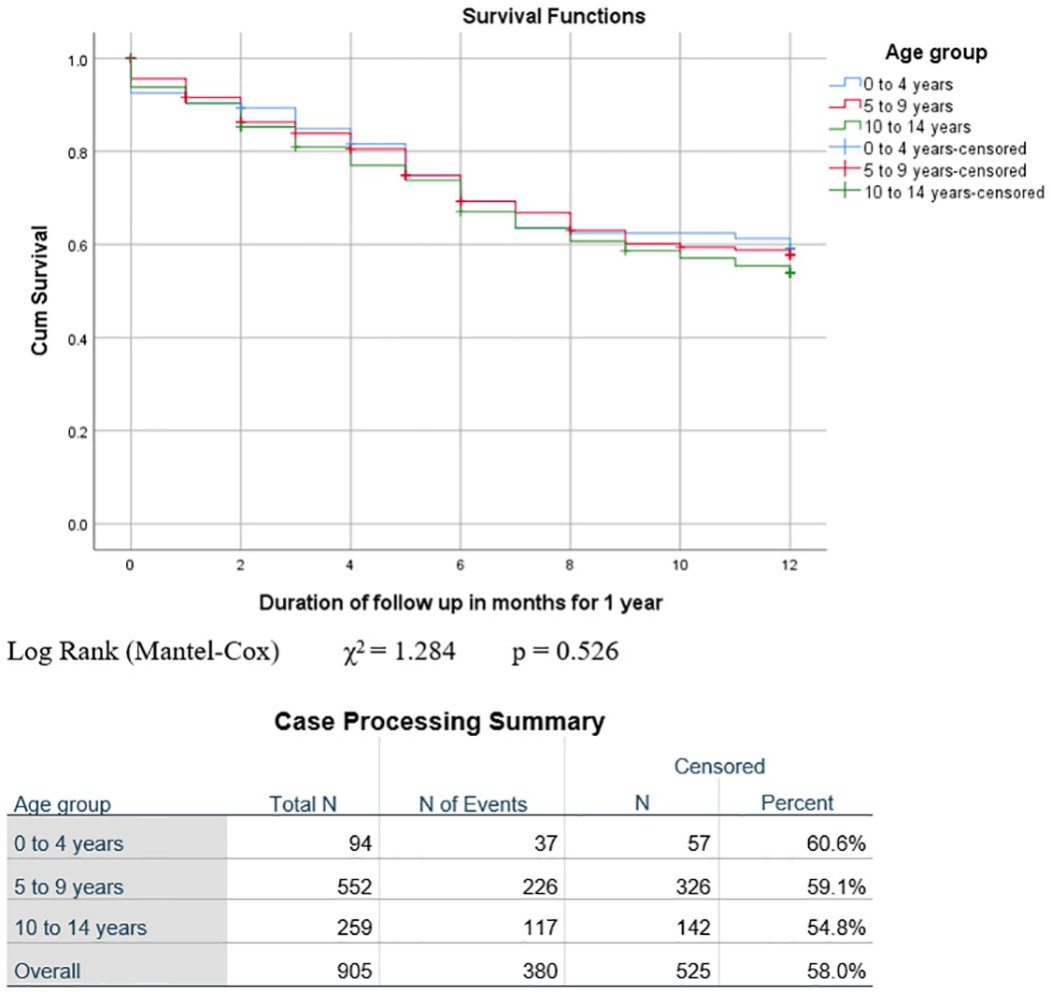
Event-free survival at one year by age group. Log Rank (Mantel-Cox) χ^2^ = 1.284 p = 0.526.

**Fig 4:**
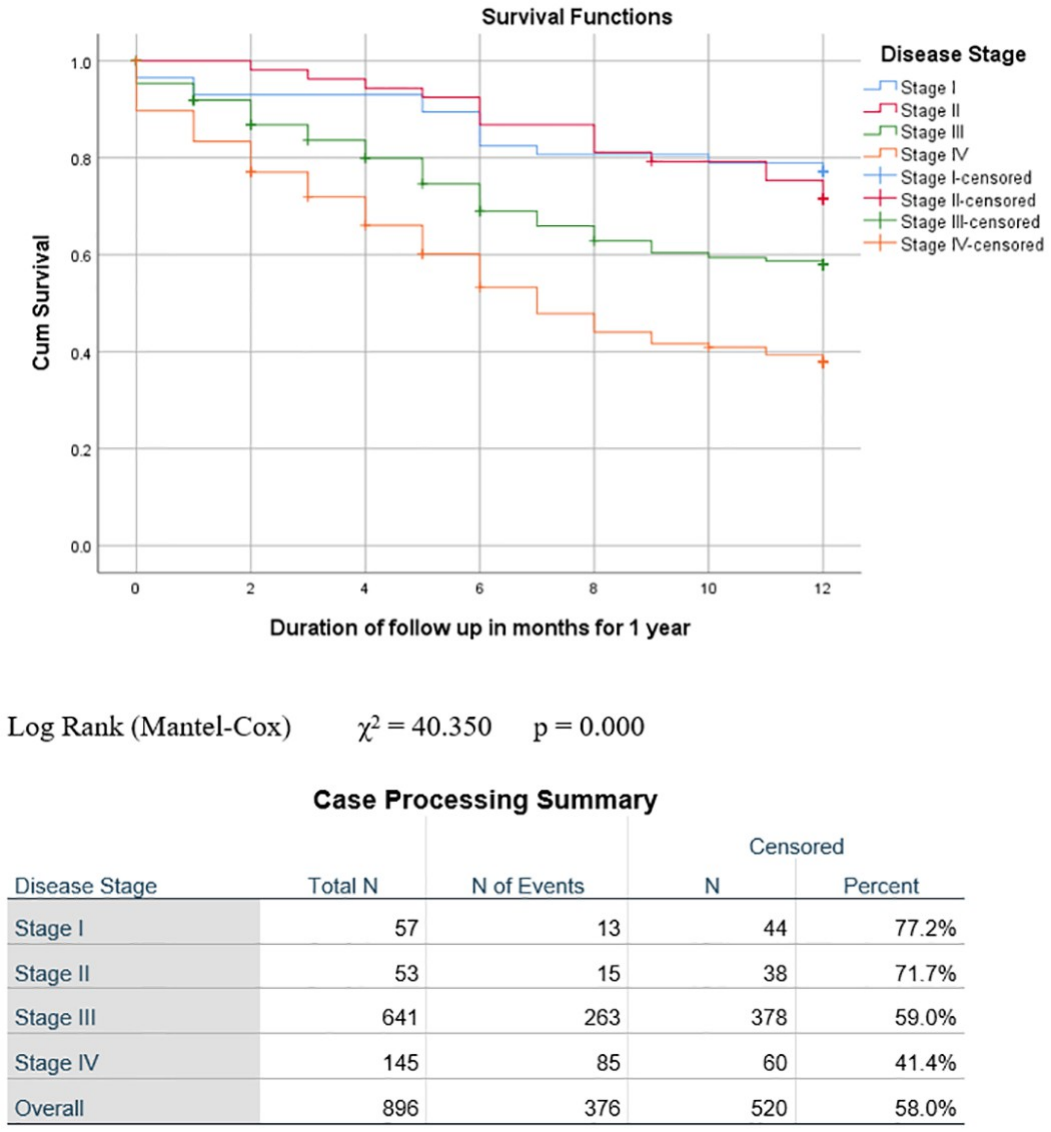
Event-free survival at one year by disease stage. Log Rank (Mantel-Cox) χ^2^ = 40.350 p = 0.000.

The outcome varied significantly by disease stage. The highest proportion of patients who were alive after one year was seen in Stage I (n = 44, 77.2%), followed by Stage II (n = 36, 67.9%) and Stage III (n = 353, 55.1%), with the lowest proportion in Stage IV (n = 47, 32.4%) (p = 0.000). Conversely, deaths were highest amongst Stage IV patients (n = 79, 54.5%), followed by Stage III (n = 212, 33.1%) and Stage II (n = 13, 24.5%), with the least number of deaths in Stage I (n = 9, 15.8%), p = 0.000. The highest proportion of deaths during treatment occurred in patients with Stage IV disease (n = 25, 17.2%), followed by Stage III (56,8.7%) and Stage I, while there were no deaths in Stage II (n = 0, 0%) (p = 0.001). Kaplan-Meier analysis of overall survival and survival by sex, age group and stage is illustrated in Figs 1–4. Patient survival decreased significantly with increasing disease stage (Spearman correlation coeff. -0.237, p = 0.000). The number of deaths during treatment and at one year both increased significantly with a higher disease stage (Spearman correlation coeff. 0.133, p = 0.000 and Pearson correlation coeff. 0.199, p = 0.000 respectively). The proportion of patients lost to follow-up was highest in Stage III (n = 51, 8%), followed by Stage I (n = 4, 7%) and Stage IV (n = 6, 4.1%), with the lowest proportion in Stage II (n = 2, 3.8%). More males (47, 8.9%) than females (16, 4.2%), (p = 0.007) were lost to follow-up (Table 2).

**Table 2:** Treatment received and outcome by disease stage.

**Treatment status/outcome (N = 896)** *	**Stage I** **n (%)**	**Stage II** **n (%)**	**Stage III** **n (%)**	**Stage IV** **n (%)**	**One-way ANOVA F (P value)**
Completed treatment	54 (94.7)	51 (96.2)	560 (87.4)	107 (73.8)	9.378 (0.000)
Died during treatment	3 (5.3)	0 (0)	56 (8.7)	25 (17.2)	5.918 (0.001)
Abandoned treatment	0 (0)	2 (3.8)	25 (3.9)	13 (9.0)	3.386 (0.018)
Alive at 1 year	44 (77.2)	36 (67.9)	353 (55.1)	47 (32.4)	15.301 (0.000)
Dead at 1 year	9 (15.8)	13 (24.5)	212 (33.1)	79 (54.5)	12.833 (0.000)
Lost to follow up	4 (7.0)	2 (3.8)	51 (8.0)	6 (4.1)	1.185 (0.314)
Delay to diagnosis in Days	**Stage I** **Median (IQR)**	**Stage II** **Median (IQR)**	**Stage III** **Median (IQR)**	**Stage IV** **Median (IQR)**	**Independent value Kruskal Wallis test p value**
Delay to diagnosis in Days	31 [20.0–101.5]	28.5 [14–61.3]	31.0 [21–91.0]	34 [21.0–90.0]	0.203

*Missing data = 934 –N.

## Discussion

This is study describes the effect of age, sex, and delay to diagnosis on the survival of Burkitt lymphoma patients in a large cohort in Cameroon. The overall treatment completion rate was 86% and one-year survival rate was 53%, both of these were only significantly affected by the disease stage. The survival rate at one year was better than the 45% recorded from Ghana [19]. Important differences in comparing the data from Ghana should be noted. The number of patients in this study is much larger. Routine cytological examination of the cerebrospinal fluid was not performed, the bone marrow was not routinely examined and routine ultrasound of the abdomen was not possible at the time. This implies that staging and survival rates by stage are not accurately comparable. All the patients in Ghana received variable amounts of cyclophosphamide and intrathecal methotrexate, but in later years cytosine arabinoside and vincristine was added to the primary treatment. In this study all patients were treated with cyclophosphamide and intrathecal methotrexate only.

The male to female ratio was 1.4:1, similar to other reports from Cameroon and other African settings [6,11,20]. The modal age group was 5–9 years (61%) which is reported to be the peak age group for BL incidence [1]. Median delay from onset of symptoms to diagnosis was 31 days. This was not significantly different between sexes nor age groups. Diagnosis delay is a problem that has been reported repeatedly amongst children with cancer in Africa and recognized as a major cause of low survival rates for childhood cancers [14]. Yaris et al. suggested that a higher incidence in childhood cancers in LMIC might be because of a culturally higher importance attached to male children than females resulting in more attention to their health problems [21]. In this study however, there was no significance in the delay to diagnosis between boys and girls nor by age group. Also, longer delay to diagnosis have been reported with other childhood cancers with up to 10 months for retinoblastoma [22,23]. Our median delay of 31 days while high considering that early diagnosis leads to better outcomes, was lower than 91 days in a report from East Africa [24]. However, in the East African study, the authors used a more systematic method to collect data treatment delay, breaking it into three periods: time-to-first health care facility contact, time-to-referral, and time-to-treatment after arrival to the cancer center [24] and might have been lower than with other cancers because Bl presents with fast growing tumours that cause pain and disfigurement with bigger tumours, causing the parents to seek medical care.

The treatment completion rate was 86%, with 9.5% deaths during treatment and 4.5% treatment abandonment. A higher rate of death during treatment has been reported for all common and curable childhood cancers across several African as well as treatment abandonment rates [25,26]. The use of SIOP recommended adapted treatment protocol for LMICs might have contributed to the manageable level of treatment toxicity hence fewer deaths during treatment and across the study period, there were varying levels of support provided by the centres for out-of-pocket costs to families, enabling them to stay in treatment [11,12]. Treatment completion was significantly affected by disease stage. This is reflected in a higher rate of death during treatment with increasing disease stage. Abandonment of treatment was also significantly more with higher disease stage. The families are largely dependent on subsistent farming and generally have several children [12]. The length of treatment and the perceived poor prognosis for children with late-stage disease evokes the dilemma of prioritizing the child’s treatment or other priorities for the livelihood of the rest of the family for these parents. Nonetheless, this issue challenges the aptitude of the clinical team in adherence counselling for the children and the parents.

The overall survival of 53% is consistent with previous reports on smaller cohorts from the same treatment centres, given that this study covers a longer period during which there were changes in the protocols [11,12,27]. Kruger et al. postulated that disease stage was the major determinant of survival for children treated for retinoblastoma in these centres with the SIOP recommended adapted protocol for low-income settings [16]. In this study, irrespective of possible cultural patterns and family priorities between sexes and ages of children as suggested elsewhere [21,28], the only factor significantly associated with survival was disease stage. This study could not confirm the finding in Ghana that boys aged between the age of six and nine years were at higher risk of relapse and death.

## Limitations

This study is a secondary analysis of hospital-based registry data. One limitation is the lack of a specific hypothesis, with multiple comparisons between variables. Also, we relied on data entered into the POND web-based database at the three centres available for running the registry also fluctuated and the capacity to make prompt and accurate diagnosis improved over time [17]. These might have affected the quality and completeness of data capturing as seen with missing data for some patients in several fields reported. We have indicated the missing data on the tables to reflect this and missing data were left out of the statistical analyses for the fields concerned. Additionally, information on patient age and delay from onset of symptoms was obtained from parents and might have been affected by recall bias. The bias is assumed to be evenly spread because the cohort consists of families with similar level of education and socioeconomic status [12].

## Conclusion

This study found that age and sex had no significant association with diagnosis or treatment delays, or abandonment, or survival rates, but younger children under five years of age presented with limited disease. However, disease stage was significantly associated with death during treatment, treatment abandonment and survival. The long delay between onset of symptoms and presentation at a health care facility, with the long time needed to confirm the diagnosis remain major challenges in our attempts to improve the survival rate.
